# Editorial: The CXCR4 Ligand/Receptor Family and the DPP4 Protease in High-Risk Cardiovascular Patients

**DOI:** 10.3389/fimmu.2016.00058

**Published:** 2016-02-19

**Authors:** Heidi Noels, Jürgen Bernhagen

**Affiliations:** ^1^Institute of Molecular Cardiovascular Research (IMCAR), RWTH Aachen, University Clinic Aachen, Aachen, Germany; ^2^Vascular Biology, Institute for Stroke and Dementia Research, Klinikum der Universität München, Ludwig-Maximilians-University, Munich, Germany; ^3^Munich Cluster for Systems Neurology (SyNergy), Munich, Germany

**Keywords:** cardiovascular diseases, diabetes mellitus, chronic kidney disease, CXCR4, CXCL12/SDF-1, MIF, CD26/DPP4, chemokine

**The Editorial on the Research Topic**

**The CXCR4 Ligand/Receptor Family and the DPP4 Protease in High-Risk Cardiovascular Patients**

Cardiovascular disease (CVD) is the most common cause of morbidity and mortality worldwide and was responsible for 17.5 million deaths in 2012. This equals 31% of all deaths globally and is almost double the amount of cancer-related deaths (1). Although CVD encompasses a broad range of pathologic conditions, 80% of all CVD-related deaths are due to heart attacks and stroke (1). An important underlying pathology is atherosclerosis, a chronic inflammatory state of the arterial wall that is characterized by lipid deposition, dysfunction of the endothelium, and infiltration of inflammatory cells into the vessel wall, resulting in the development of atherosclerotic lesions (2).

Type 2 diabetes mellitus (T2DM) and chronic kidney disease (CKD) have been identified as important risk factors for CVD. Fifty percent of patients with CKD stages 4–5 suffer from CVD (3), and cardiovascular mortality accounts for ~40–50% of all deaths in these patients, compared with 26% in controls with normal kidney function (4, 5). Also in patients with diabetes, CVD accounts for at least 50% of deaths (6). With currently around 10–13% of people presenting with CKD and more than 8% of adults suffering from diabetes (6–8), the social and economic burden of diabetes, CKD, and CVD is extremely high. Thus, a better understanding of the mechanisms contributing to and mediating the interplay between T2DM, CKD, and CVD is required to improve the prevention and treatment of these diseases.

In this *Research Topic*, we focus on the classical CXC chemokine receptor CXCR4 (9), its cognate ligand CXCL12 (10), and the chemokine-like cytokine macrophage migration inhibitory factor (MIF), which functions as a non-cognate ligand of CXCR4 (11), in the context of CVD. An emphasis is made on links with T2DM and CKD. Also, we discuss dipeptidyl peptidase-4 (DPP4) as an important protease known to destabilize CXCL12 and thus to influence signaling through the CXCL12/CXCR4 axis. Chemokines and their receptors are important mediators of cell mobilization, recruitment and arrest, and additionally more broadly induce cell activation by triggering various intracellular signaling tracks. Chemokines control basic homeostatic conditions but are also critically involved in inflammatory processes, e.g., in atherosclerosis (12, 13). Genome-wide association studies revealed single nucleotide polymorphisms connecting CXCL12 as well as MIF with CVD (2, 14–19), and a role for both of these mediators in T2DM and CKD has been reported. In this *Research Topic*, we introduce the reader to the comorbidities T2DM and CKD and their connection with CVD, and provide up-to-date information on the involvement of CXCL12/MIF/CXCR4 and DPP4 in each of these pathologies. One focus is laid upon providing insight on mechanistic level.

## T2DM, CKD, and CVD: Comorbidities Interlinked on Mechanistic Level

There is clear-cut epidemiologic evidence linking the comorbidities T2DM and CKD with CVD. Progress has also been made to explain the comorbid status of these diseases on molecular level; however, the interaction seems highly complex and insight into the connecting mechanisms is mostly still in its infancy. Here, Schuett et al. (Germany) present an overview of the inflammatory processes underlying atherosclerosis and highlight the role of inflammation in T2DM and chronic inflammatory diseases in relation to CVD. The current prevalence and incidence of T2DM, CKD, and CVD are summarized by Gajjala et al. (Germany). They also provide insight into molecular mechanisms interconnecting these comorbidities.

Animal models allow us to investigate these interconnecting mechanisms in more detail and enable us to evaluate potential therapeutic strategies. Hewitson et al. (Australia) summarize available animal models for investigating mutual interactions between cardiac and renal injury and critically discuss how representative they are for human disease. This also includes an overview of animal models for hypertension, diabetes, and obesity, which are linked with kidney and cardiac injury through systemic alterations.

## The CXCR4 Chemokine Receptor and Its Ligands CXCL12 and MIF: Molecular Aspects and Involvement in CVD, T2DM, and CKD

Pawig et al. (Germany) present the diversity and interconnections in the CXCR4 receptor/ligand family. Signaling pathways initiated by binding of CXCL12 vs. MIF to CXCR4 are discussed, and it is elaborated on how ACKR3 (previously called CXCR7) affects CXCR4 signaling. Finally, authors summarize the (patho)biological functions of CXCR4 signaling mediated by CXCL12 or MIF that are likely to be important in devising potential future therapies targeting this signaling axis.

van der Vorst et al. (Germany) present an overview of the role of CXCL12 vs. MIF in CVD, highlighting the differences and similarities. Vidakovic et al. (Serbia) discuss the controversial role of the CXCL12/CXCR4 axis in diabetes, whereas Morrison and Kleemann (The Netherlands) summarize the role of MIF in obesity, insulin resistance, T2DM, and associated hepatic comorbidities as revealed by both human and animal studies. Bruchfeld (Sweden) discusses MIF in the context of kidney disease, while complementarily, Valiño-Rivas et al. (Spain) elaborate on the expression and role of CD74, an additional receptor for MIF and the MIF homolog MIF-2 (or D-DT) in kidney injury.

## DPP4 as Regulator of CXCL12 and Its Involvement in CVD, T2DM, and CKD

DPP4 (also known as CD26) is known to destabilize CXCL12. Therefore, our Research Topic also addresses molecular aspects and functions of this protease as well as its role in CVD, T2DM, and CKD. Waumans et al. (Belgium) provide a comprehensive insight into DPP4 and its role in the immune system and inflammatory diseases including atherosclerosis. This also includes an overview of other family members of the dipeptidyl peptidase family as well as of prolyl oligopeptidases and prolyl carboxypeptidases. Circulating DPP4 is increased in patients with T2DM, and DPP4 inhibition is used for treatment of T2DM. Thus, Röhrborn et al. (Germany) provide detailed insight into the expression, enzymatic activity, and function of DPP4 in the context of diabetes, based on *in vitro*, animal, and human studies. Also, mechanistic insight into the role of DPP4 in the T2DM-associated morbidities CVD and liver disease is provided. The role of DPP4 and its regulation of the CXCL12/CXCR4 axis in CVD are then discussed in more detail by Zhong and Rajagopalan (USA). Finally, Panchapakesan and Pollock (Australia) summarize potential renoprotective effects of DPP4 inhibitors in diabetic kidney disease and also discuss the cardiovascular safety profile of DPP4 inhibitors.

Altogether, this *Research Topic* aims to assist both specialists and interested non-specialists and to stimulate further initiatives to unravel the mechanistic involvement of the CXCR4 ligand/receptor family in the detrimental interplay between the comorbidities T2DM, CKD, and CVD, potentially paving the way for new therapeutic initiatives in the future.

## Author Contributions

All authors listed, have made substantial, direct and intellectual contribution to the work, and approved it for publication.

## Conflict of Interest Statement

The authors declare that this work was conducted in the absence of any commercial or financial relationships that could present a potential conflict of interest.
